# Prescribing and Monitoring of Valproate

**DOI:** 10.1192/bjo.2026.11851

**Published:** 2026-06-30

**Authors:** Juwayriya Sullayman, Deborah Okonji

**Affiliations:** 1 Bradford Disctrict Care NHS Foundation Trust, Bradford, United Kingdom; 2 Sheffield Health Partnership University NHS Foundation Trust, Sheffield, United Kingdom

## Abstract

**Aims:**

This audit evaluates the prescribing and monitoring practices of sodium valproate, particularly in females of childbearing potential. Sodium valproate is a medicine that is commonly used in the treatment of bipolar disorder and epilepsy, and less commonly migraine prophylaxis. Valproate is an effective treatment for bipolar disorder, however there are significant risks that come with the use of valproate medication including the risk of teratogenic effects should pregnancy occur.

**Methods:**

The audit aims to assess compliance with the Pregnancy Prevention Programme (PPP), share findings ahead of the June 2025 national audit, and highlight updated initiation steps post-April 2024.

The audit standards were derived from national guidelines including the Royal College of Psychiatrists CR210 and MHRA Prevent Programme. They are also a replica of the standards measured in the National POMH Valproate Audit. The purpose of auditing the standards is to review compliance prior to the next POMH Valproate audit which will take place in June 2025.

The audit reviewed documentation of prescribing rationale, annual reviews (therapeutic benefit, adverse effects, medication adherence), and PPP compliance including Annual Risk Acknowledgement Forms (ARAFs).

The sample included 22 female patients aged 12–55 currently prescribed valproate. These patients were identified by the Clinical Director of Pharmacy. Sample characteristics such as age and ethnicity were also explored.

**Results:**

• Rationale for prescribing was documented in 20 out of 22 cases (91%).

• Only 23% of patients had all elements of the annual review documented.

• ARAF compliance was poor:

- 9 non-compliant cases where pregnancy was possible.

- 10 non-compliant cases where pregnancy was not possible.

• Common issues included missing or incorrectly completed ARAFs and lack of documentation of Patient Guide distribution.

**Conclusion:**

The audit revealed significant gaps in compliance with PPP requirements and documentation standards. There was confusion around when ARAFs are needed, especially for long-standing patients and those post-menopausal. Baseline checks prior to initiation (BMI, FBC, LFT) were not consistently documented. The findings suggest a need for improved education and communication among prescribers.

The audit highlights the need for better adherence to national guidelines in prescribing and monitoring valproate. Key areas for improvement include annual review documentation, proper completion of ARAFs, and baseline physical health checks. Recommendations include targeted education for consultants and improved documentation practices to ensure patient safety and regulatory compliance.

